# Economic inequality in malnutrition: a global systematic review and meta-analysis

**DOI:** 10.1136/bmjgh-2021-006906

**Published:** 2021-12-09

**Authors:** Rotimi Alao, Hayaan Nur, Emily Fivian, Bhavani Shankar, Suneetha Kadiyala, Helen Harris-Fry

**Affiliations:** 1 Department of Population Health, London School of Hygiene & Tropical Medicine, London, UK; 2 Department of Geography, The University of Sheffield, Sheffield, UK

**Keywords:** systematic review, nutrition

## Abstract

**Objective:**

To describe the evidence on global and regional economic inequality in malnutrition, and the associations between economic inequality and malnutrition.

**Methods:**

We conducted a systematic review and meta-analysis. Between 1 November 2020 and 22 January 2021, we searched Medline, Embase, Global Health, Eldis, Web of Science and EBSCO Discovery Service. We contacted 39 experts and tracked citations. We included any study reporting a concentration index (CIX) relating economic status and nutritional status and any multilevel study reporting an association between economic inequality and nutritional status. Nutritional status was measured as stunting, wasting, anaemia, or overweight in children (<5 years), or underweight, overweight or obesity, or anaemia in adults (15–49 years). We had no study date or language restriction. Quality was assessed using the Appraisal Tool for Cross-Sectional Studies (AXIS tool). We mapped estimates and pooled them using multilevel random-effects meta-analyses.

**Results:**

From 6185 results, 91 studies provided 426 CIX (>2.9 million people) and 47 associations (~3.9 million people). Stunting (CIX −0.15 (95% CI −0.19 to −0.11)) and wasting (−0.03 (95% CI −0.05 to −0.02)) are concentrated among poor households. Adult overweight and obesity is concentrated in wealthier households (0.08 (95% CI −0.00 to 0.17)), particularly in South Asia (0.26 (95% CI 0.19 to 0.34)), but not in Europe and Central Asia (−0.02 (95% CI −0.08 to 0.05)) or North America (−0.04 (95% CI −0.10 to 0.03)). We found no association between 0.1 increase in Gini coefficient and adult underweight (OR 1.03 (95% CI 0.94 to 1.12)) or overweight and obesity (0.92 (95% CI 0.80 to 1.05)).

**Conclusions:**

There is good evidence that the prevalence of malnutrition varies by levels of absolute economic status. Undernutrition is concentrated in poor households, whereas concentration of overweight and obesity by economic status depends on region, and we lack information on economic inequalities in anaemia and child overweight. In contrast, links between malnutrition and relative economic status are less clear and should not be assumed; robust evidence on causal pathways is needed.

**PROSPERO registration number:**

CRD42020201572.

**Key questions**

**What is already known?**

- Progress in improving nutrition is being hampered by economic inequalities.
- Several individual studies have characterised economic inequalities in nutrition, but we lack understanding of where inequalities are largest and where evidence gaps are.
- Reviews show positive associations between measures of economic inequality and health outcomes, but none have reviewed associations with nutritional status.

**What are the new findings?**

- Child undernutrition is concentrated among poor households, and this is not explained by country-level measures of income, food security or healthcare coverage.
- Adult overweight and obesity is concentrated in better-off households globally, but this is explained by evidence from lower-income countries and not higher-income countries.
- Associations between economic inequality and nutrition outcomes show no clear overall trend.
- There are large gaps in evidence on anaemia and child overweight, which is concerning given the lack of progress on these global targets.

**What do the new findings imply?**

- Our findings highlight the need for pro-poor targeting in undernutrition interventions, whereas the level and direction of targeting for interventions on overweight and obesity will need to be determined by context.
- Although countries’ economies, food systems and healthcare systems protect against undernutrition, work is needed to ensure they benefit those who need it most.

## Introduction

No country is on track to meet all World Health Assembly global nutrition targets by 2025.^1^ One in nine people is hungry or undernourished, while one in three is overweight or obese,^1^ and there are wide cross-country disparities. Undernutrition rates are up to 10 times higher in lower-income countries, while overweight and obesity are up to five times higher in higher-income countries.^1^ Furthermore, national-level statistics mask wide socioeconomic disparities within countries. There is now consensus that further progress in reducing the burden of malnutrition worldwide will require targeted action to address these within-country inequalities.^1 2^

A growing but disparate literature has sought to characterise economic inequalities in malnutrition in different countries, using metrics from economics: concentration curves (CCs) and concentration indices (CIXs).^3–5^ CCs plot the cumulative proportion of a health outcome in a population against the cumulative proportion of the population ranked in ascending order of economic status, and CIX are twice the area between the CC and line of equality (the 45° line from the origin). Rather than simply compare the richest against the poorest, CC and CIX capture the inequality across the full study population, and CC also visualise where the biggest health burdens lie.^4 6^ However, this effort has not been systematically mapped and individual estimates are difficult to interpret in isolation. We lack an understanding of where and why economic inequalities in nutritional status are largest.

Furthermore, these economic inequalities in nutrition outcomes often lead to the assumption that relative economic inequality—rather than absolute economic status—causes malnutrition. Economic inequality in society could plausibly harm nutritional status through different pathways.^7–10^ These include direct and indirect pathophysiological effects of social comparisons^11–13^ and effects on appetite, diets, breastfeeding behaviour and physical activity; social exclusion^14–17^ and effects on access to health services and other entitlements; and disinvestment in human development, for example, in public education, food systems and sanitation infrastructure.^18–20^ Reviews on the effects of income inequality on population health more generally (using aggregate outcomes such as mortality and life expectancy) show that the effect of inequality is heterogeneous and often harmful to health,^21–23^ but the effects on malnutrition are less understood.

The latest Global Nutrition Report^1^ highlighted a need to better understand inequalities in nutrition, to inform priority setting, redress inequalities and aid countries to meet global targets of eliminating world hunger and reversing the rise in overweight and obesity. Our study responds to this call with two study aims, to systematically review evidence that: (1) characterises economic inequality in malnutrition of adults and children worldwide, and (2) estimates the association between economic inequality and malnutrition.

## Methods

### Search strategy and selection criteria

Our systematic review and meta-analyses followed a registered protocol^24^ and Preferred Reporting Items for Systematic Reviews and Meta-Analyses guidelines.^25^ The study population was children aged <5 years or adults aged 15–49 years. Outcomes were chosen because they align with World Health Assembly Global Nutrition Targets.^1 26^ For children, outcomes were stunting (height-for-age z-score SD <-2 of WHO child growth standards median), wasting (weight-for-height z-score SD <-2), anaemia (haemoglobin <110 g/L) and overweight (weight-for-height SD >2). For adults, outcomes were underweight (low body mass index (BMI) <18.5 kg/m^2^), overweight or obesity (BMI >25 kg/m^2^) and anaemia (pregnant women <110 g/L; non-pregnant women <120 g/L; men <130 g/L).

Our exposures were inequalities in economic status, where economic status was measured as household-level wealth, total expenditure or income. We excluded studies that used an incomplete measure of economic status, such as land size or food expenditures, or measured economic status at the individual level. We considered the household as an economic unit, because individual-level income data are not routinely collected or meaningful for young children. For adults, household wealth enables us to rank subjects without misclassifying household members who benefit from household economic status but lack personal income or wealth, although there is some risk of misclassification due to intrahousehold inequalities in income or wealth allocation.^27^

First, to characterise economic inequality in nutrition outcomes, we included evidence using any study design that reported a CC or CIX.^3^ CC and CIX were chosen based on an initial scoping search and dissertation by author (RA)^5^ that determined these to be the most common metric to capture inequality across the full wealth distribution. As mentioned, CCs plot the cumulative proportion of a health outcome in a population against the cumulative proportion of the population ranked in ascending order of economic status. The CC lies above or below the line of equality (the 45° line from the origin) if the outcome is more prevalent among the poorer or richer households respectively.^4 6 28 29^ The CC may also intersect the line of equality, which would mean that households at that point in the wealth distribution have exactly their proportionate level or prevalence of the health outcome, and those to the left and right of the intersection have proportionately more and less of the outcome respectively (or vice versa). The CIX is twice the area between the CC and the line of equality. Negative (positive) CIX values indicate the health outcome is more concentrated among poor (rich) households.^4 6 18 28 29^ We converted CIX to the Erreygers version,^30^ which corrects for dependence on the mean of the outcome but retains the same qualitative interpretation.

Second, to review the association between economic inequality and nutrition outcomes, we included multilevel studies where households were nested within larger geographical areas, and the exposure was the Gini coefficient. Gini coefficients were also chosen because our scoping research found this to be the most common measure of income inequality. Gini coefficients are based on Lorenz curves, which are similar to CC except they depict economic inequality rather than health inequality.^31^ This means that they plot the cumulative proportion of economic status (rather than a health outcome) against the cumulative proportion of the population ranked by economic status. Analogous to CC and CIX, the Gini coefficient is twice the area between Lorenz curve and the 45° line of equality. Values closer to 0 or 1 indicate lower or higher inequality, respectively. A key difference between Lorenz curves and CC is that the former must always be below line of equality whereas the CC can be above or below it, and this means that Gini coeffficients are bounded by 0 and 1, whereas CIX are bounded by −1 and +1. This is because health outcomes can be concentrated among the poor, while wealth, by definition, cannot.

We only included multilevel studies because they permit the inclusion of household-level economic status, so can disentangle effects of economic inequality from absolute economic status,^32^ and are at lower risk of bias from ecological fallacy.^32 33^

We searched Medline, Embase, Global Health, Eldis, Web of Science and EBSCO Discovery Service between 1 November 2020 and 3 November 2020. EBSCO Discovery Service includes the National Bureau of Economic Research, EconLit, Jstor and Scopus. We used keywords for ‘wealth or income,’ ‘inequality,’ ‘concentration index’ or ‘multilevel models,’ and, ‘nutritional status’ (sample search string in online supplemental table S1). There were no date or language restrictions. We contacted 39 experts and tracked citations of relevant reviews and articles included for full-text screening.

10.1136/bmjgh-2021-006906.supp1 Supplementary data

Two reviewers (HN and RA) doubly screened the articles, first by title and abstract, and then by full text. Disagreements were resolved by a third reviewer (EF).

### Data extraction

Data were doubly extracted by HN, RA, EF and HH-F. We extracted data on sample characteristics; sample size; response rate; outcome indicator and prevalence; measure of economic status; data source and year; geographical unit; measure of inequality; effect sizes that could be converted into CIX or logit estimates; measures of variance that could be converted into 95% CIs; covariates; author names; survey date and publication year. When estimates were disaggregated by obesity and overweight, or by gender, we extracted the aggregated results if presented, and otherwise used the disaggregated estimates. We contacted authors to retrieve missing data on outcome prevalence and SEs or where interpretations needed clarification. CCs were extracted with the web plot digitiser online tool.^34^ We handled duplicates (estimates on the same outcome from the same survey data) by selecting estimates with the highest quality rating and averaging the remaining duplicates. For maps, we handled duplicates in the same way but only included latest and nationally representative estimates.

### Quality assessment

The quality of evidence was assessed using the Appraisal Tool for Cross-Sectional Studies (AXIS tool) at the study level.^35^ Two reviewers independently graded each included study as ‘low’, ‘moderate’, ‘high’ or ‘critical’ risk of bias. Grades are given in online supplemental table S2, S4A. Discrepancies in overall ratings were discussed and resolved. We excluded no studies based on quality but conducted subgroup analyses to assess if study quality unduly affected our conclusions.

### Data analysis

We conducted meta-analyses and mapped CIX for child stunting, wasting and adult overweight or obesity; other outcomes produced ≤2 studies so were not included in a meta-analysis or mapped but are narratively described. For associations between Gini coefficient and nutritional outcomes, results were standardised to logit estimates and meta-analyses were possible for adult underweight, and overweight and obesity. To account for the hierarchical structure of the data (several estimates per country and region), all meta-analyses were performed as multilevel random effects meta-analyses.^36^

We cleaned and plotted CCs with Stata (V.16.1), ran multilevel models with R (V.4.0.3) and created maps using ArcMap (V.10.8.1).

We report Q statistic to describe heterogeneity in study estimates. Funnel plots and Egger tests assessed publication bias.

Prespecified subgroup analyses were: study quality rating, tertiles of country-level estimates of food security (dietary energy supply, measured as kcal/capita/d),^37^ country income level (low, lower-middle, upper-middle and high gross national income, as per World Bank classification),^38^ tertiles of WHO universal health coverage score (scale of 0–100, measured as the mean of 14 indicators),^39^ seven World Bank geographical regions, and tertiles of study date. Additionally, for associations between Gini coefficient and nutrition outcomes, we disaggregated by the geographical unit the Gini coefficient was measured (first, second and third and lower subnational levels) as this was suggested as an important mitigating factor.^40 41^

## Results

**Figure 1:**
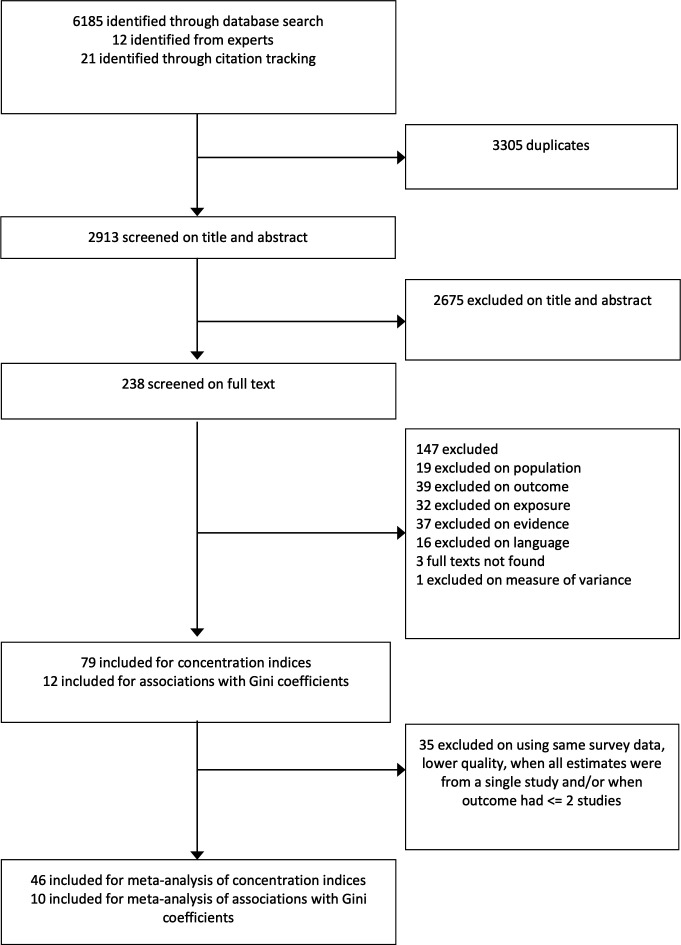
Study selection.

Figure 1 shows the study selection process. We included 79 articles reporting economic inequalities in malnutrition outcomes using CIX.^29 42–119^ Of these, 46 studies (426 estimates) were included in meta-analyses, giving 277 estimates (90 countries) for stunting, 60 (33 countries) for wasting and 89 (21 countries) for adult overweight or obesity.

A summary of study characteristics for CIX included in meta-analysis is given in online supplemental table S2. Most estimates were from sub-Saharan Africa for stunting (47%) and wasting (50%). For overweight and obesity, most estimates were from Europe and Central Asia (49%).

**Figure 2:**
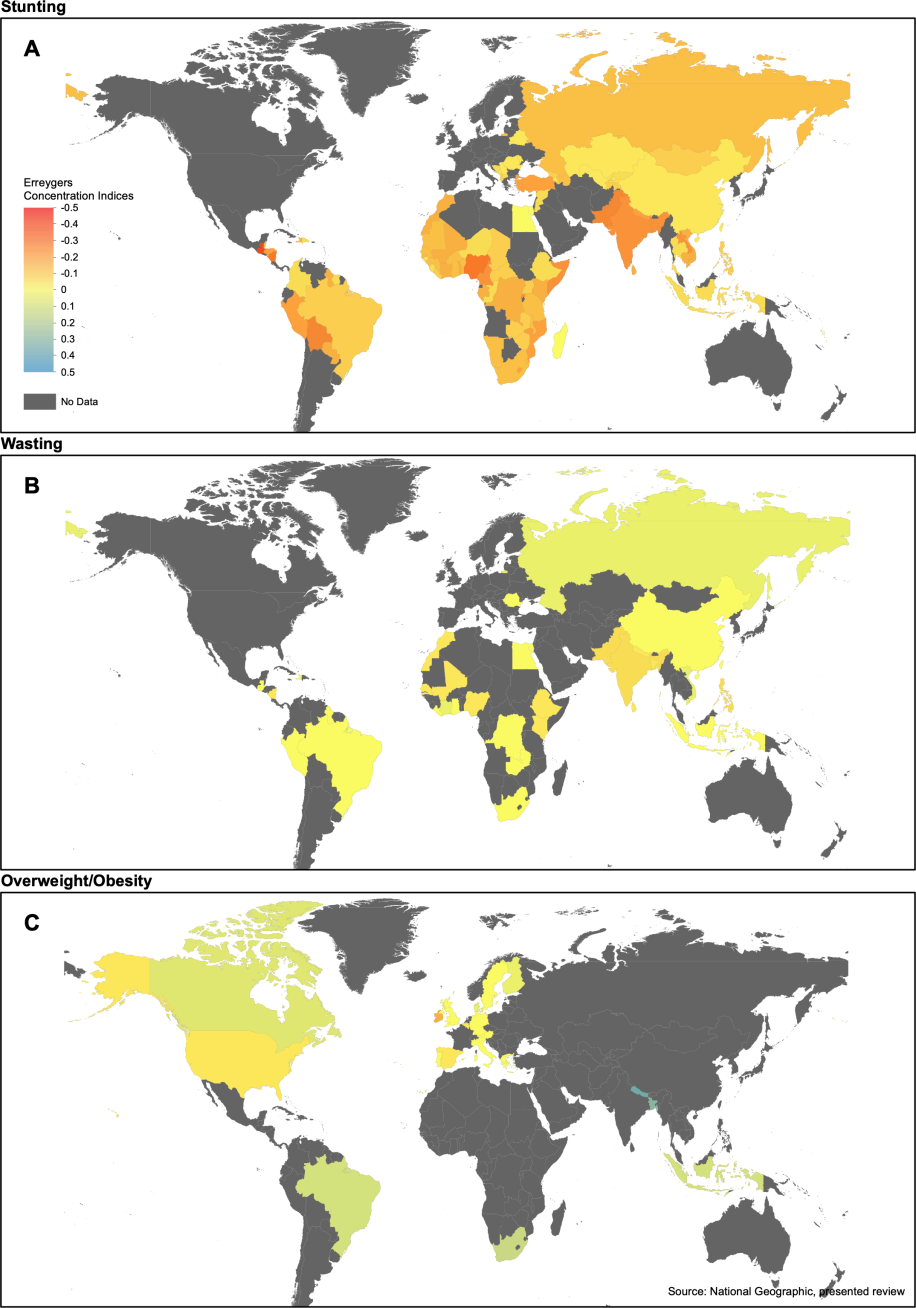
Map of most recent concentration indices for prevalence of stunting, wasting and overweight and obesity.

Figure 2A–C maps nationally representative CIX for stunting (88 countries), wasting (30 countries) and adult overweight and obesity (19 countries). They show a lack of evidence on the concentration of stunting or wasting by economic status in some African and Southern Asian countries, where stunting and wasting remains prevalent but CIX have not been published, and very few CIX for overweight and obesity published across Africa, Asia and Latin America.

**Figure 3:**
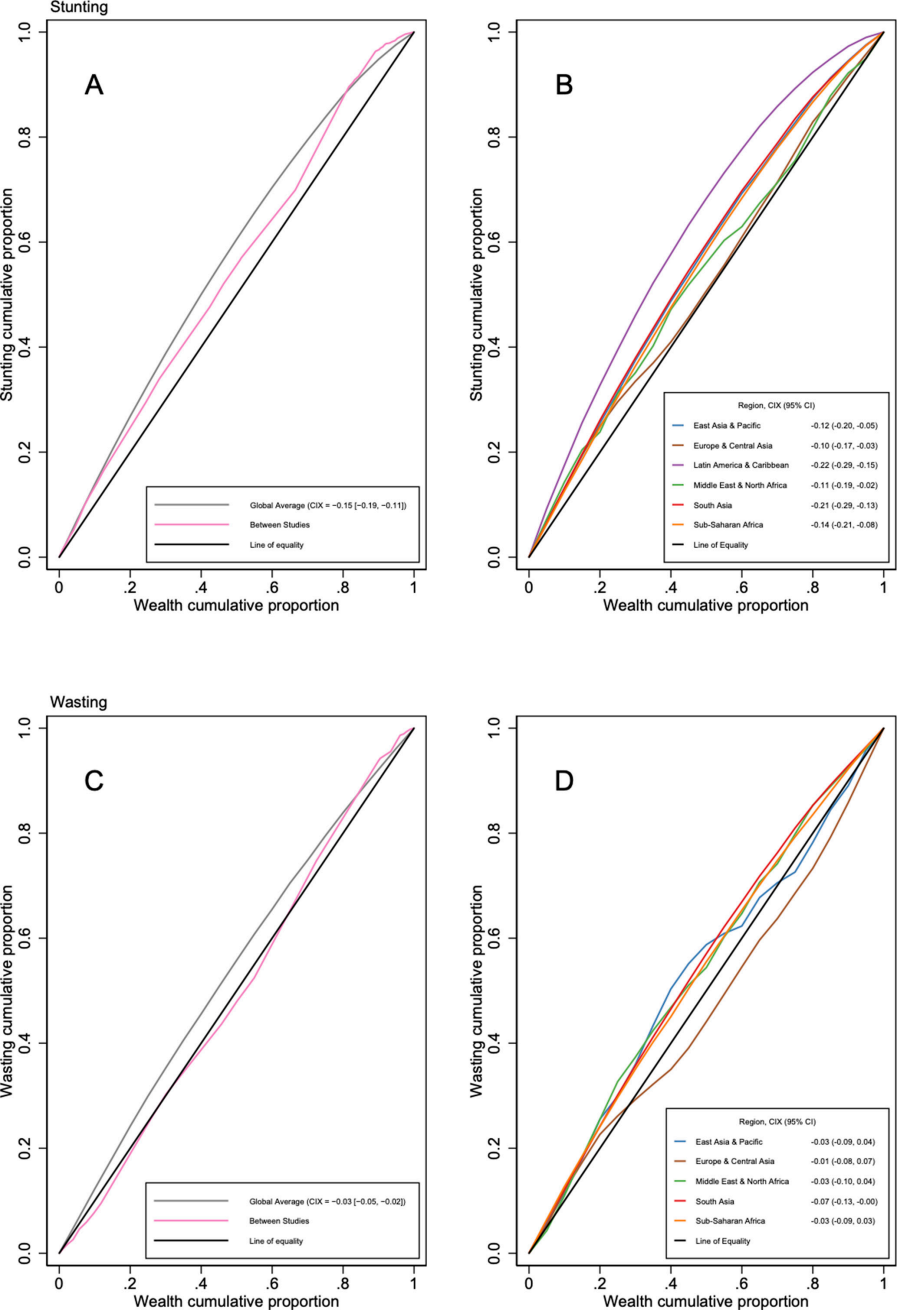
Concentration of stunting and wasting by economic status. Average concentration curves plot population-weighted average cumulative proportion of malnourished subjects over the population-weighted average cumulative proportion of economic status. Between studies concentration curve plot cumulative product of population and prevalence over the cumulative Gross Domestic Product of countries. CIX, concentration index.

Figure 3A shows the pooled CIX estimate for stunting of −0.15 (95% CI −0.19 to –0.11), and a global average CC which bulges towards the upper left of the plot and is strictly above the equality line, indicating concentration of stunting among poor households within studies. Stunting is more concentrated within than between studies, except for the richest 20% of the population and the richest 20% of study contexts. There is high overall heterogeneity (Q=9299, p<0.01) (online supplemental figure S1). Individual CCs (online supplemental figure S2) confirm virtually all estimates find stunting concentrated in poorer households.

Figure 3B shows the inequality in stunting is most pronounced in Latin America and the Caribbean (CIX −0.22 (95% CI −0.29 to –0.15)) and South Asia (−0.21 (95% CI –0.29 to –0.13)). The map of most recent stunting estimates in figure 2A supports this and illustrates that, despite the relatively narrow CI, the Latin America and Caribbean region still contains large levels of heterogeneity, with some of the most and least extreme concentrations (eg, Guatemala (2008) with CIX −0.42 vs Brazil (2006) with CIX −0.03). The map also indicates consistently low CIX in the most recent estimates for South Asia, and higher CIX in some Eastern European countries, Brazil, China, Egypt and Madagascar.

Figure 3C shows wasting is also concentrated among poor households (pooled CIX −0.03 (95% CI −0.05 to –0.02), and it is also more concentrated within than between studies. Estimates are also heterogeneous (Q=446; p<0.01) (online supplemental figure S3). The regionally disaggregated curves (figure 3D) show wasting is concentrated among the poor in all regions, with smaller differences between regions than stunting. There are slightly higher CIX in South Asia, and lower CIX in Europe and Central Asia (the latter due to estimates from Armenia and Thailand, as shown in online supplemental figure S4).

**Figure 4:**
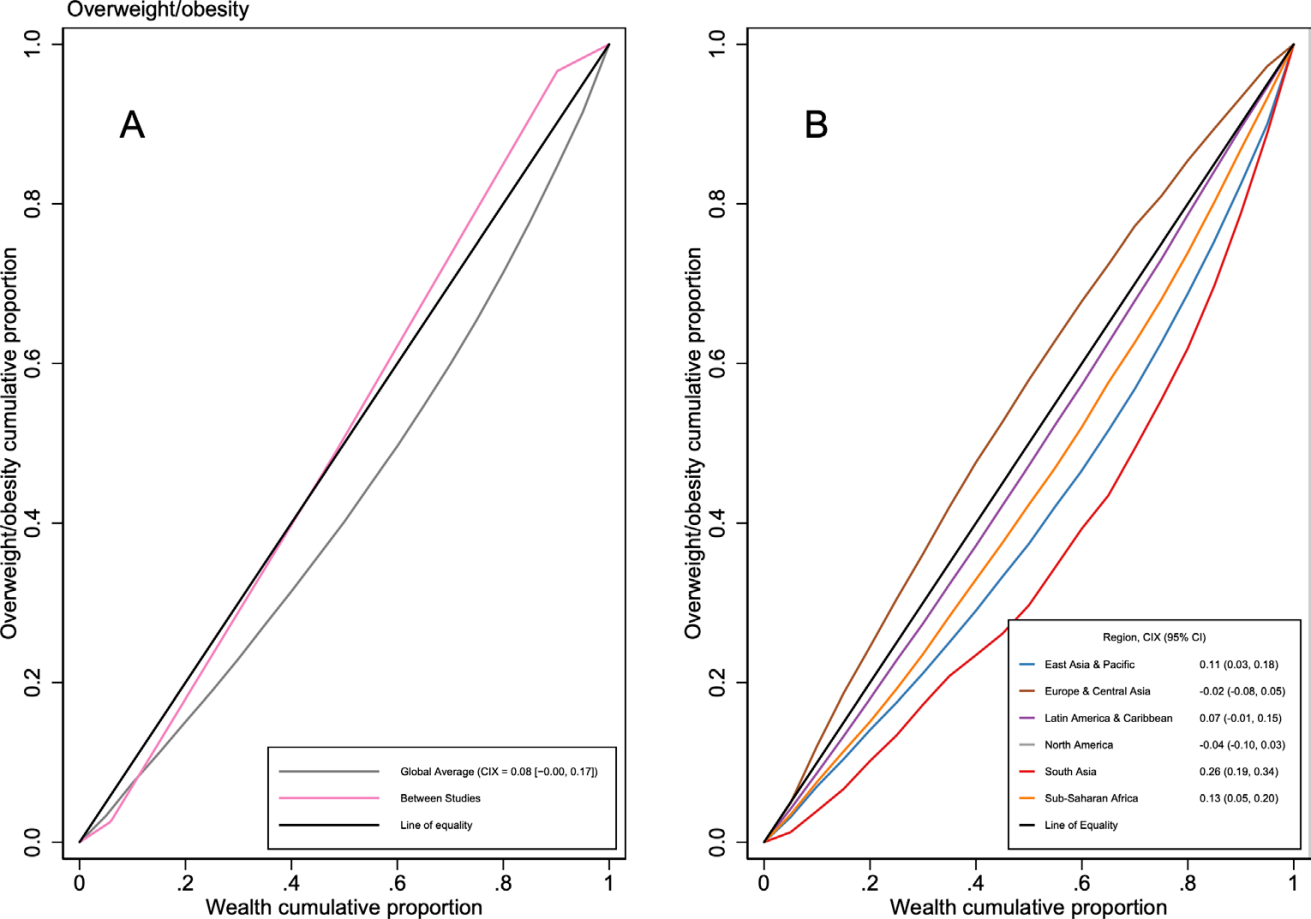
Concentration of overweight and obesity by economic status. Average concentration curves plot population-weighted average cumulative proportion of malnourished subjects over the population-weighted average cumulative proportion of economic status. Between studies concentration curve plot cumulative product of population and prevalence over the cumulative Gross Domestic Product of countries. No concentration curves available for North America. CIX, concentration index.

Figure 4A shows overweight and obesity is slightly more concentrated among better-off households (CIX 0.08 (95% CI −0.00, 0.17)) and there is also more inequality within than between studies. Results are heterogeneous (Q=34 324; p<0.01) (online supplemental figure S5).

**Figure 5:**
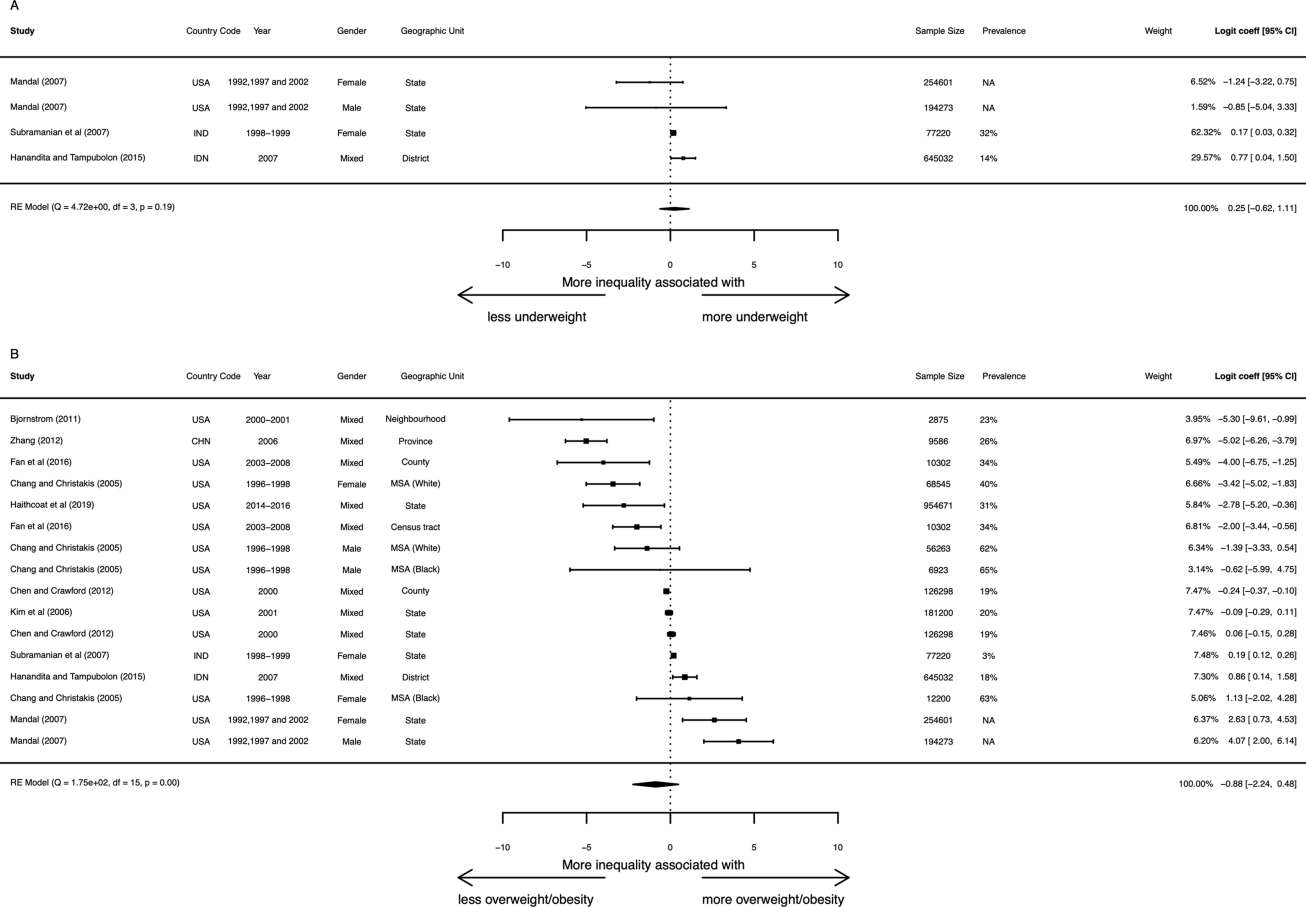
Meta-analysis of association between economic inequality and malnutrition. Random effects meta-analysis adjusting for clustering within countries and regions, where exposure (Gini coefficient) is on a 0–1 scale, and effect size is logged odds of malnutrition. MSA (black)—individuals of black ethnicity in Metropolitan Statistical Areas, MSA (white)—individuals of white ethnicity in MSA. MSA, metropolitan statistical areas.

Regional subgroups (figure 4B) show more concentration of overweight and obesity among better-off households in studies from South Asia (0.26 (95% CI 0.19 to 0.34)), sub-Saharan Africa (0.13 (95% CI 0.05 to 0.20)) and East Asia and Pacific (0.11 (95% CI 0.03 to 0.18)), and comparatively more concentration among poorer households in North America (−0.04 (95% CI −0.10 to 0.03)) and Europe and Central Asia (−0.02 (95% CI −0.08 to 0.05)). Figure 2C and online supplemental figure S6 show these pooled CIX estimates are driven by higher CIX in studies from Nepal, Bangladesh, Indonesia and a multicountry study from Africa, and lower CIX in Canada, USA and western Europe. Consistent with this, subgroup analyses show that overweight and obesity is concentrated among better-off households in studies from low-income and lower-middle-income countries (0.18 (95% CI 0.09 to 0.28) for both) (online supplemental figure S5).

Aside from the subgroups mentioned, other subgroups for stunting, wasting, or overweight and obesity do not show consistent trends (online supplemental figures S1, S3 and S5, respectively).

For outcomes not meta-analysed, we found two studies each on child anaemia, adult anaemia, and adult underweight, and one for child overweight. For child anaemia, one study showed moderate concentration of anaemia among poor households across 35 countries in sub-Saharan Africa −0.10 (95% CI −0.11 to –0.10),^83^ and the other showed the same in Cambodia, reported as CC.^105^ For anaemia in adults, one study showed small CIXs in Nepal (range: −0.05 to 0.02)^93^ and another showed larger CIX in Bangladesh (−0.14 (95% CI −0.17 to –0.11)).^79^ For adult underweight, studies also showed a smaller CIX from Nepal (−0.04 (95% CI −0.07 to –0.00))^94^ and a larger CIX from Bangladesh (−0.23 (95% CI −0.24 to –0.21).(75) For child overweight we found a small CIX from households in 35 sub-Saharan African countries (CIX 0.01 (95% CI 0.01 to 0.02)).^83^

Assessing within-study bias, we classified most CIX studies as moderate risk (44/79; 56%), but this proportion was lower in estimates included in meta-analysis due to removal of duplicates with higher risk of bias (online supplemental table 2). The most common limitation was risk of misclassifying economic status. Subgroups analyses found no clear differences by risk of bias. Egger tests and funnel plots (online supplemental table S3 and figure S7) indicate effect sizes and heterogeneity may be exaggerated by small-study effects for stunting and overweight and obesity, but not wasting.

To review the association between Gini coefficient and nutrition outcomes, we identified 12 studies (47 estimates)^40 41 120–129^ (online supplemental table S4A). Of these, 10 were included in meta-analyses. We identified 6 estimates for stunting, 6 for adult underweight (4 independent), 33 for adult overweight and obesity (16 independent), 1 each on adult and child anaemia, and none on wasting. Authors’ covariate adjustments are given in online supplemental table S4B.

Child stunting estimates emanate from the same study on two countries: Bangladesh and Kenya.^41^ Effect sizes range from highly negative (logit: −2.53 (95% CI −4.40 to –0.65)) to highly positive (logit: 3.84 (95% CI 0.28 to 7.41)).

For adult underweight, estimates come from three studies in USA, India and Indonesia. Figure 5A shows no consistent association with the Gini coefficient (logit: 0.25 (95% CI −0.62 to 1.11); four estimates). In terms of ORs, this is 1.03 (95% CI 0.94 to 1.12). Subgroup analyses show no clear differences (online supplemental figure S8).

For adult overweight and obesity (figure 5B), we find no consistent association with Gini coefficient (logit: −0.88 (95% CI −2.24 to 0.48); 16 estimates). OR is 0.92 (95% CI 0.80 to 1.05). Effects are heterogeneous (Q=175, p<0.01), and most of the precise estimates are close to zero, whereas estimates showing positive or negative associations are less precise. Subgroup analyses show no consistent differences (online supplemental figure S9).

For anaemia, we found one study, which showed higher odds of children having anaemia if they lived in a country with higher inequality (logit coefficient 0.61 (95% CI 0.34 to 0.88); 30 countries) but no difference for anaemia in women (0.16 (95% CI −0.06 to 0.38); 33 countries).^122^

We classified most (9/12, 75%) associational studies as moderate risk of bias. Common limitations were unclear measurement of outcome or exposure, and not justifying the sample size. Risk of bias did not explain the heterogeneity of associations in the subgroup analyses. There is some indication of small-study effects for adult overweight and obesity but not adult underweight (online supplemental table S5 and figure S10).

## Discussion

Our review found that almost all CIX for child undernutrition were negative, indicating that child undernutrition is disproportionately concentrated in the poorest households worldwide. In contrast, the direction of CIX in overweight and obesity varies regionally. In some places, like South and East Asia and sub-Saharan Africa, overweight and obesity are concentrated among better off households, while in North America and Europe and Central Asia, if anything, poorer households are more affected. We also find important evidence gaps, with a lack of studies reporting CIX for child overweight, and adult and child anaemia. Although these CIX show wide inequalities in the distribution of malnutrition, we find overall null associations between our measure of income inequality (Gini coefficients) and nutritional status. Evidence on the links between economic inequality and undernutrition is too thin to draw robust conclusions, and heavily biased towards the USA for adult overweight and obesity. We need globally representative research to unpack causal pathways between economic inequality and the different forms of malnutrition.

Our study has some limitations. First, reviews of observational studies carry a risk of bias. We find some evidence of bias within and between studies, as shown by some potential small-study effects, perhaps due to the lack of methodological standardisation. Second, to minimise risk of bias, we restricted our review of associations with Gini coefficients to multilevel studies. This may produce conservative estimates and have narrower geographical coverage. However, ecological studies we discuss below find a similarly heterogeneous picture. Third, all studies were published before the COVID-19 pandemic; country-specific estimates may need to be updated after such a large shock to both health and economies.

Our review shows that, while still a global problem,^130^ undernutrition is most common in poor households in low-income countries. These results are consistent with other studies that only look at the extremes of the distribution or compare wealth groups across countries.^1^ The concentration of stunting is also regionally clustered, perhaps because neighbouring countries share common factors that affect nutrition and economic status, such as agroecological conditions, structural barriers, food environments, dietary behaviours, sanitation, education and employment levels.^131–133^

Given that almost every undernutrition CIX in our review was negative, isolated studies producing another negative CIX provide limited additional value. Our global and regional CIX give context for future inequality studies, so magnitudes can be compared against our estimates. These results also highlight the need for undernutrition interventions to be ‘pro-poor’, for example, by providing targeted interventions (such as social safety nets) and ensuring universal interventions have equitable reach and quality (such as universal healthcare).^1 134^ However, none of the country-level factors that we thought might modify the relationship between economic status and nutrition—income level, food security and healthcare coverage—explain economic inequalities in undernutrition. Therefore, although they protect against undernutrition,^135 136^ work is needed to ensure countries’ economies, food systems, and health systems benefit those who need it most.

In contrast, we find more concentration of overweight and obesity in better-off households from lower-income countries compared with high-income countries. These findings are consistent with a systematic review,^137^ which showed that the association between economic status and obesity varies by country income level; our review adds to this by describing and mapping the extent of the concentration within and between study populations. Our findings also align with the broader evidence base on the global nutrition transition, whereby overweight and obesity (historically more common in richer households from higher-income countries) is now more common in poorer households in higher-income countries and in better-off households in lower-income countries.^138 139^ However, our subgroup analyses (online supplemental figures S1, S3, S5) do not reveal clear time trends, and small cell sizes do not permit exploration of heterogeneity in time trends between higher-income and lower-income countries.

Nevertheless, our results indicate that the level and direction of targeting of overweight and obesity interventions will need to be contextually specific. Depending on the intervention and country, it may be useful to target better-off households from lower-income countries (eg, social and behaviour change interventions to discourage unhealthy food selection in supermarkets and retail outlets frequented by the well-off)^140^ or poorer households in higher-income countries (eg, providing healthy free school meals).^141^ However, to comprehensively address the multilevel drivers of all forms of malnutrition, it is increasingly recognised that macrolevel interventions across sectors and countries are needed to increase individual’s agency, and create healthy food environments and equitable food systems.^1 142^

Despite large economic inequalities in the burden of malnutrition, and previous studies showing that economic inequality is associated with poor health,^21–23^ we found little overall support that economic inequality is associated with malnutrition. We lack evidence on the associations between Gini coefficients and undernutrition, limiting our ability to draw robust conclusions. As noted, evidence on overweight and obesity is disproportionately from the USA. Although significant trends may be observed in other countries, an ecological study of 31 OECD countries found that the positive correlation between economic inequality and obesity disappeared after excluding the USA and Mexico, suggesting this may not be the case.^143^ Ecological studies also show wide heterogeneity, finding that economic inequality is associated with higher,^144 145^ lower^146^ or no difference^147^ in overweight or obesity. It is difficult to discern whether these inconsistent trends are due to the difficulties in identifying unbiased estimates or variation in true effects. A further complication is that associations between Gini coefficients and nutritional outcomes may be non-linear, and could interact with absolute levels of economic status. Although all studies included in our review adjusted for household-level income or wealth status, they predominantly focused on linear associations between economic inequality and nutrition, and were not designed to compare the effects of absolute vs relative income or wealth on malnutrition. Future research could include wider geographical contexts, include different forms of malnutrition, and characterise the nature of the relationship between absolute and relative income or wealth.

## Data Availability

No data are available.
