# Supplementary material for: Economic inequality in malnutrition: a global systematic review and meta-analysis
Source: BMJ Glob Health. 2021 Dec 9;6(12):e006906. doi: 10.1136/bmjgh-2021-006906 (PMC8663078; doi:10.1136/bmjgh-2021-006906)
Supplement: Supplementary data [file bmjgh-2021-006906supp001.pdf]

Supplementary material

Table S1: Medline search strategy

1	Wealth* or asset* or income* or earning* or poor* or povert* or depriv* or economic status or economic inequality or expenditure or equit* or equal* or inequal* or dispar*
2	Exp poverty/ or exp health equity/ or exp economic status/ or exp health status disparities/
3	Underweight or overweight or adipos* or BMI or body mass index or (body-mass adj2 ind*) or obes* or weight-for-height or weight-for-length or WHZ or WLZ or stunt* or HAZ or height-for-age or length-for-age or wasting or wasted or an?emi* or h?emoglob* or undernutri* or overnutri* or undernour* or overnour* or malnutri* or malnour* or thinness or overweight* or underweight* or nutritional status
4	Exp thinness/ or exp overweight/ or exp anemia/ or exp nutrition disorders/ or exp obesity/ or exp body mass index/
5	(Concentration* adj3 curve*) or (concentration adj3 ind*) or (Lorenz adj3 curve*)
6	(1 or 2) and (3 or 4) and 5
7	((Wealth* or income* or earning* or poor* or povert* or economic) adj3 (inequ* or equ* or distribut* or disparit*)) or gini or theil or pietra or schutz or Robin Hood-ind* or attkinson or (decile* adj3 ratio*) or inequality
8	Multilevel or multi-level or (two adj3 level*) or two-level or random effects or random effect or random-effect or random-effects or (control* adj5 individual*) or (mixed adj3 effect*) or mixed-effect or mixed-effects or nested data or random coefficient or random coefficients or (hierarchic* adj3 (model* or data*))
9	(3 or 4) and 7 and 8
10	6 or 9

Table S2: Characteristics of studies included in the meta-analysis of concentration indices

	Child stunting, number of estimates (N) =277		Child wasting, N=60		Adult overweight /obesity, N=89	
	**Mean or %**	**(Min, max)**	**Mean or %**	**(Min, max)**	**Mean or %**	**(Min, max)**
Survey date, mean year	2004	(1974, 2018)	2003	(1987, 2018)	2001	(1971, 2016)
% estimates measuring income	8.3%	..	33.3%	..	83.1%	..
% estimates measuring wealth	91.0%	..	63.3%	..	16.9%	..
% estimates measuring expenditure	0.7%	..	3.3%	..	..	..
Mean outcome prevalence	32.5%	(4.0%, 61.0%)	10.1%	(1.0%, 25.0%)	22.1%	(4.4%, 88.2%)
Mean concentration index	-0.164	(-0.441, 0.027)	-0.038	(-0.174, 0.018)	-0.012	(-0.280, 0.339)
WHO universal health coverage index	54.8	(22, 78)	51.6	(35, 78)	78.2	(46, 88)
FAO Food security index	108.1	(25, 155)	108.6	(82, 141)	133.9	(102, 149)
Low-income country, %	53.6%	..	56.7%		5.6%	
Region, %	..	..	..	..	..	..
East Asia & Pacific	6.1%	..	6.7%	..	5.6%	..
Europe & Central Asia	10.8%	..	3.3%	..	49.4%	..
Latin America & Caribbean	21.3%	..	11.7%	..	4.5%	..
Middle East & North Africa	5.4%	..	5%	..	0%	..
South Asia	8.7%	..	23.3%	..	5.6%	..
Sub-Saharan Africa	46.7%	..	50.0%	..	5.6%	..
North America	0%	..	0%	..	29.2%	..
Gender composition of sample	..	..	..	..	..	..
Mixed	100%	..	100%	..	30.3%	..
Male only	0%	..	0%	..	31.5%	..
Female only	0%	..	0%	..	38.2%	..
Risk of bias						
Low	0%		0%		7.9%	
Moderate	91.0%		91.7%		70.8%	
High	8.7%		6.7%		16.9%	
Critical	0.4%		1.7%		4.5%	

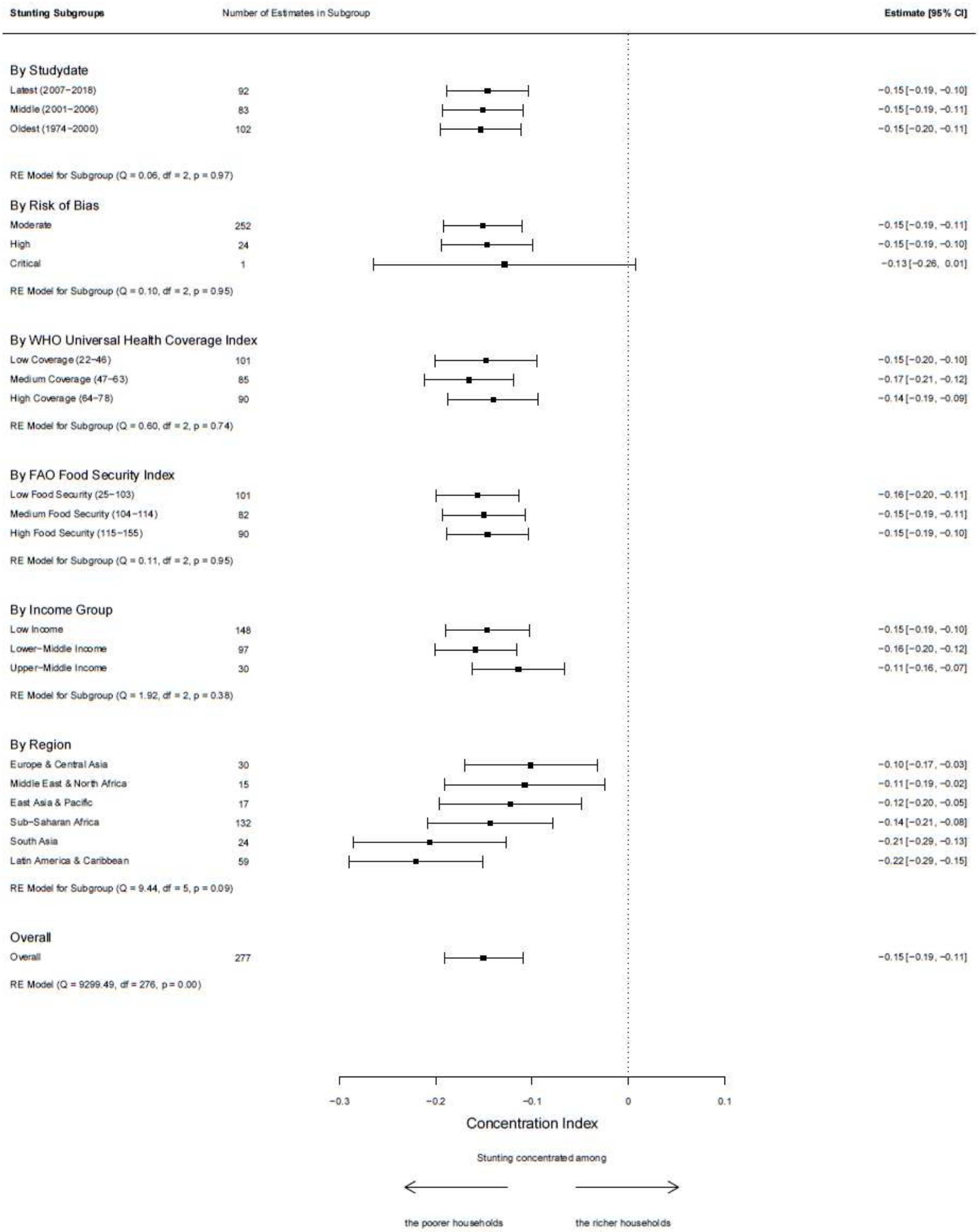

Figure S1: Subgroup analysis for concentration indices of stunting

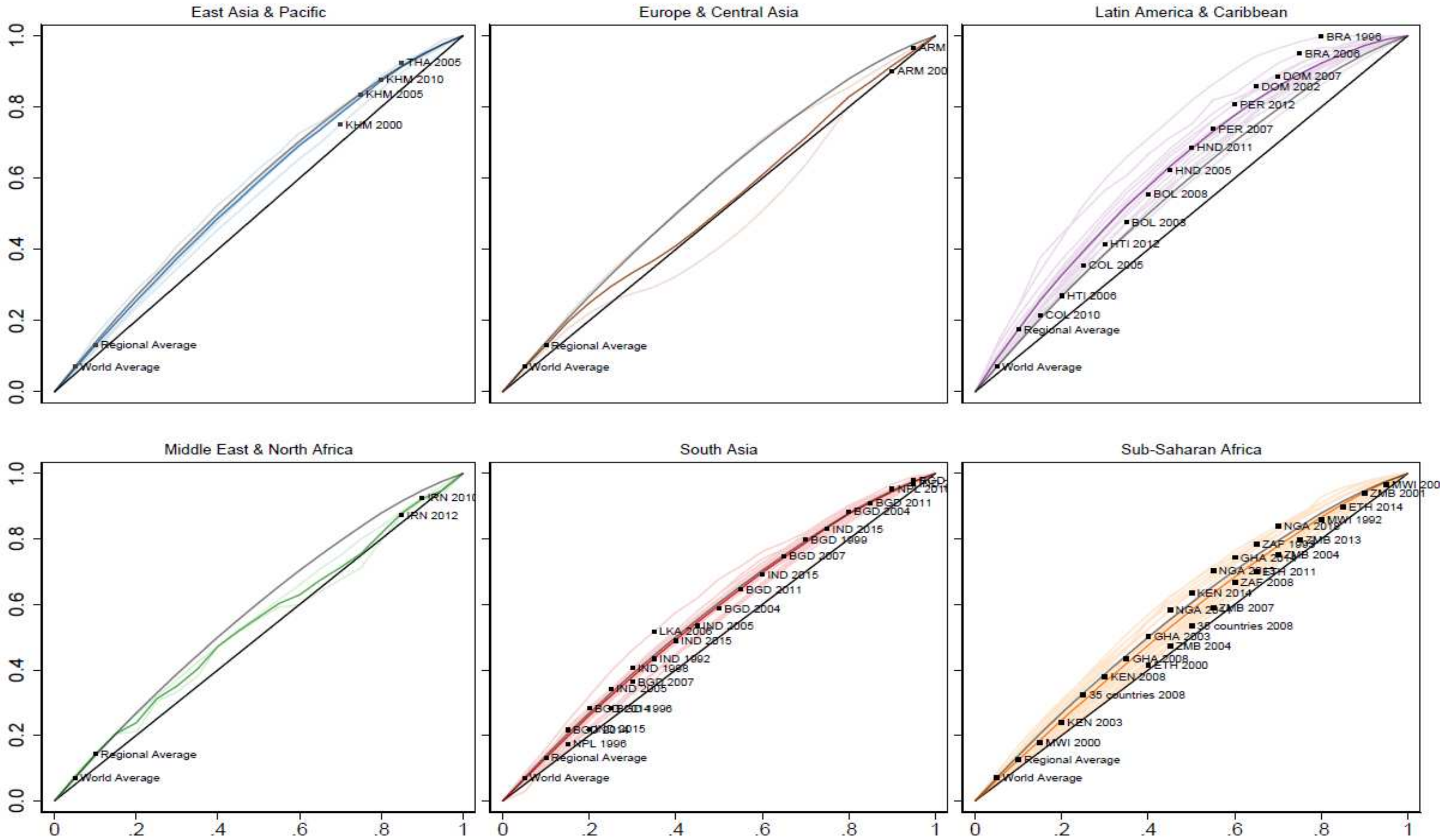

Figure S2: Regional stunting concentration curves with country names

Cumulative proportion of wealth on the X-axis. Cumulative proportion of stunting on the Y-axis. Earliest year of data collection and three-letter ISO country code call-outs.

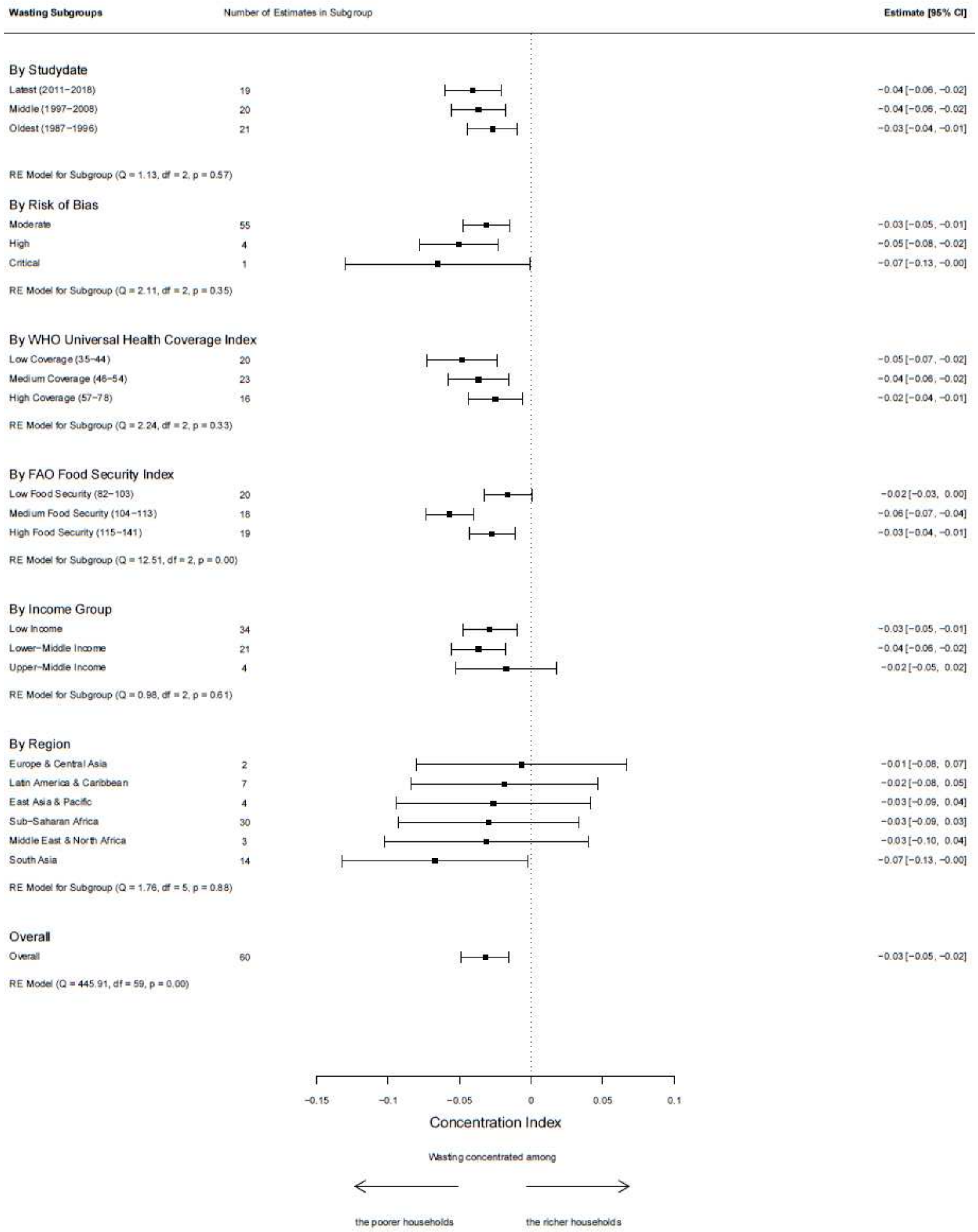

Figure S3: Subgroup analysis for concentration indices of wasting

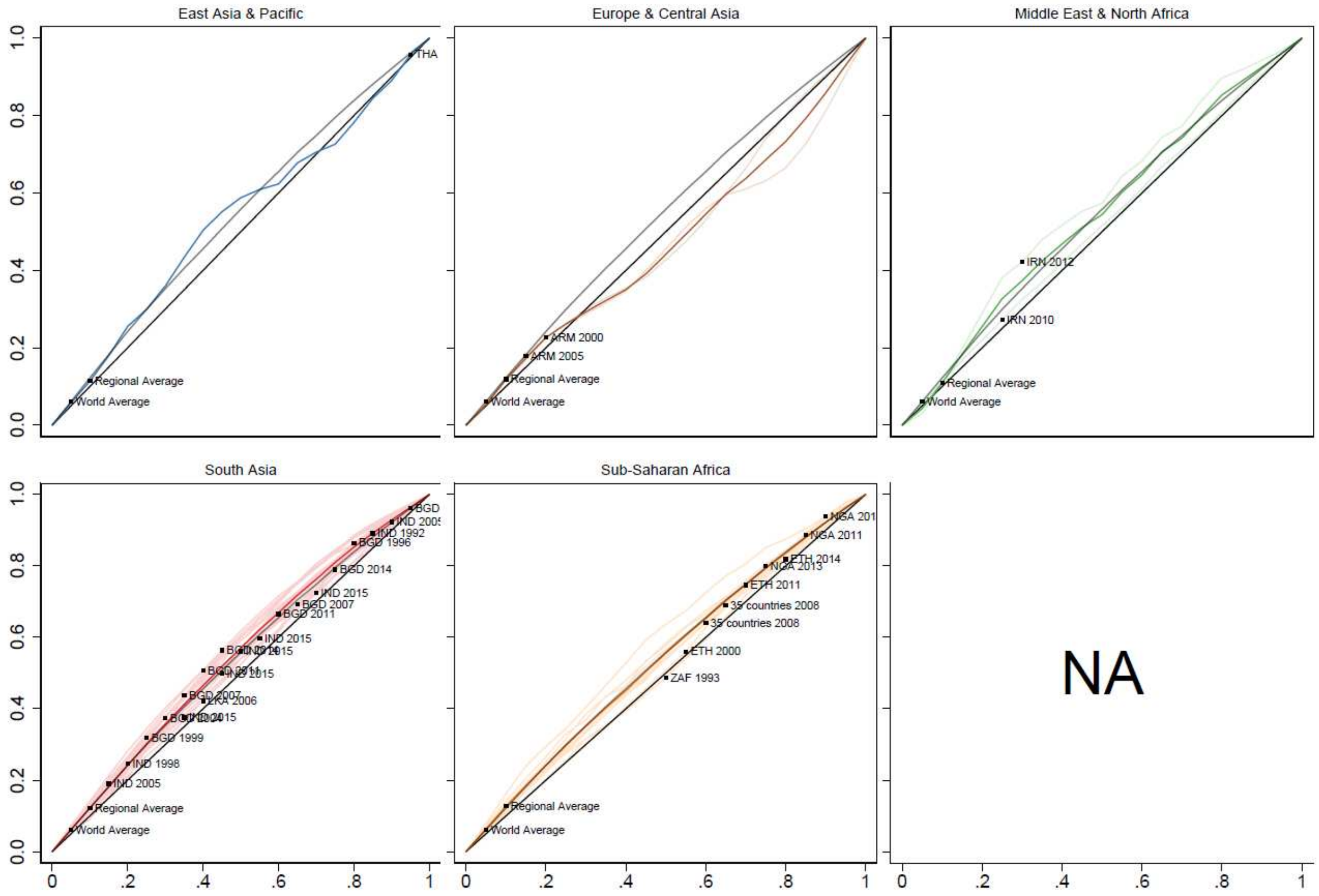

Figure S4: Regional wasting concentration curves with country names

Cumulative proportion of wealth on the X-axis. Cumulative proportion of stunting on the Y-axis. Earliest year of data collection and three-letter ISO country code call-outs.

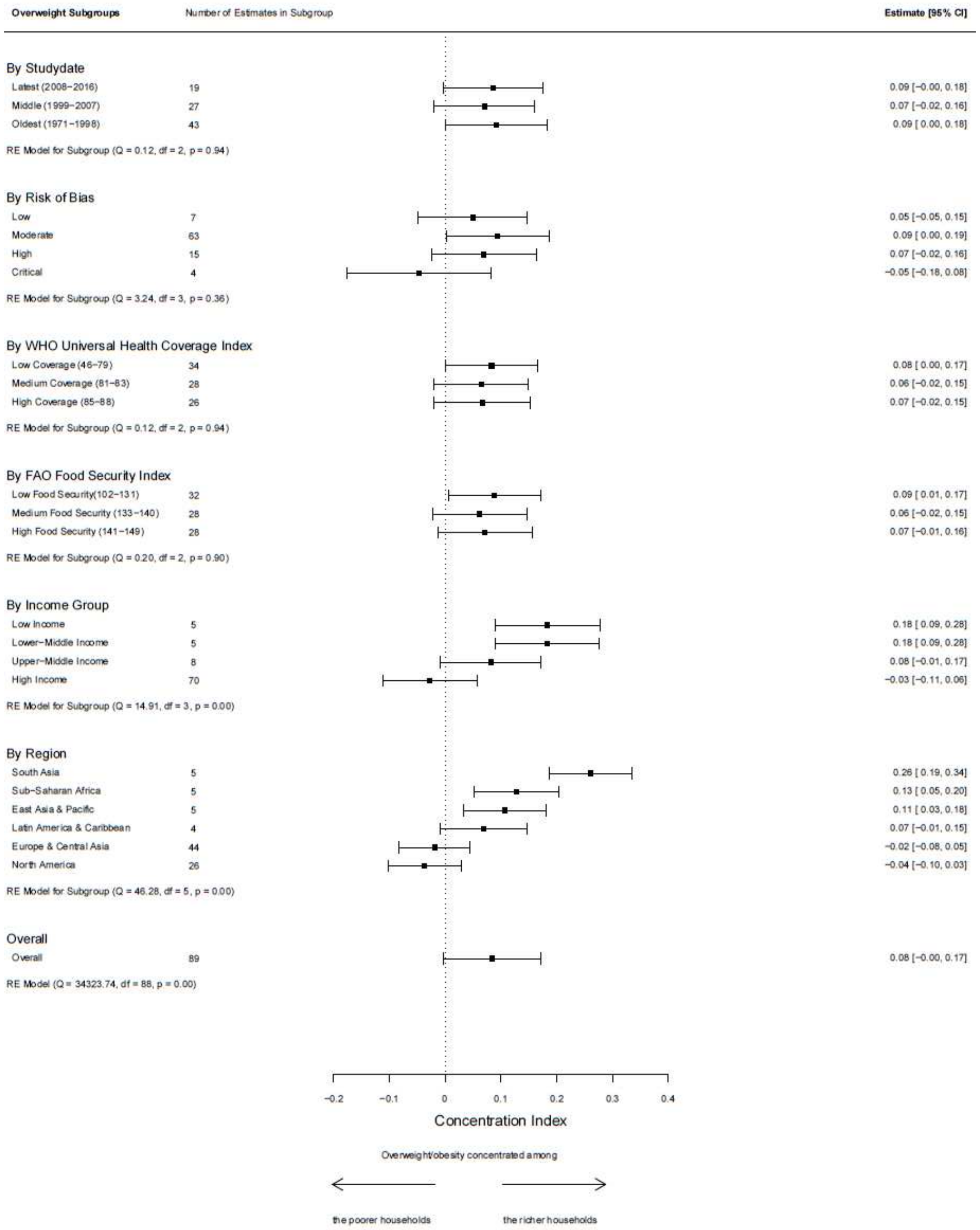

Figure S5: Subgroup analysis for concentration indices of adult overweight/obesity

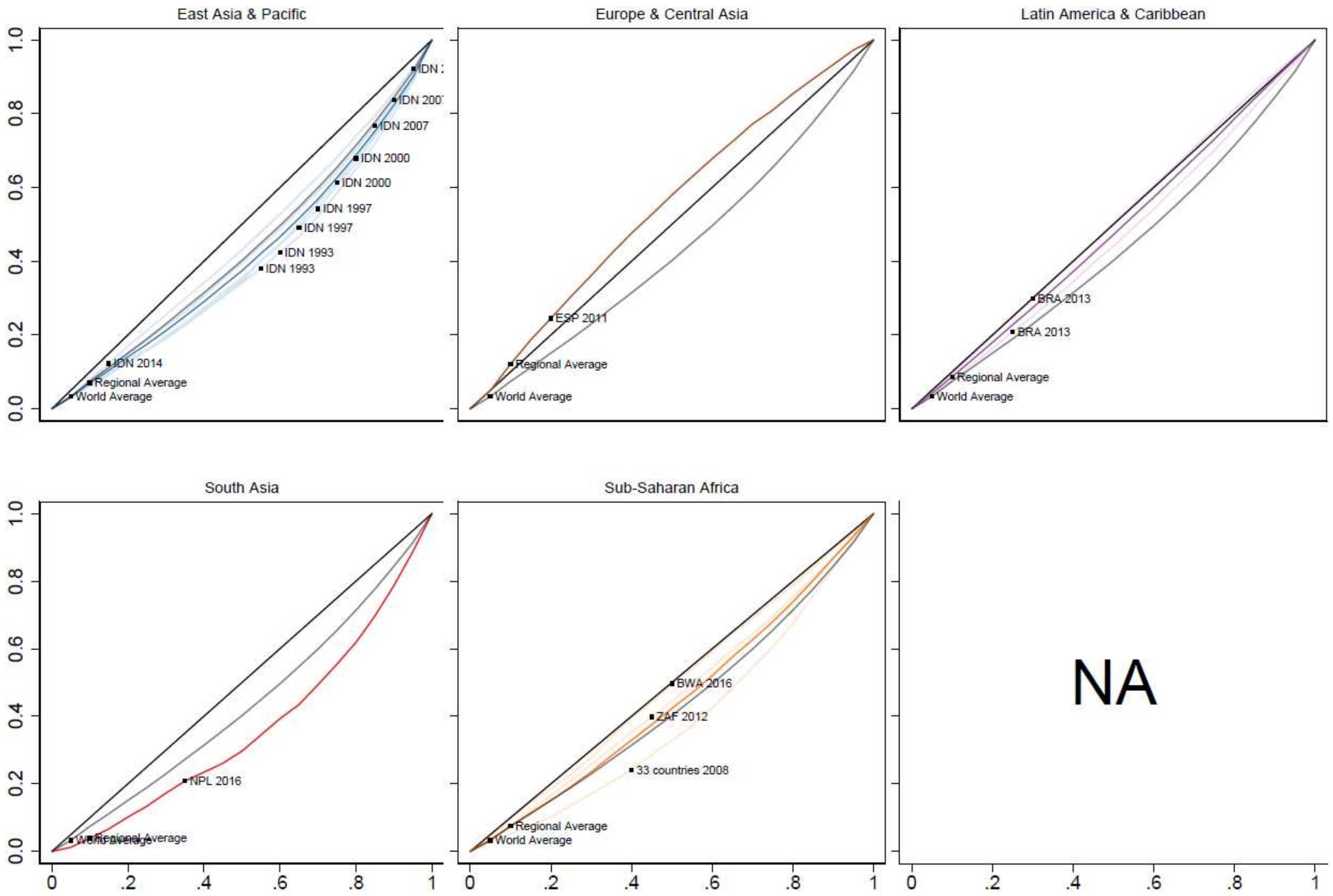

Figure S6: Regional overweight/obesity concentration curves with country names

Cumulative proportion of wealth on the X-axis. Cumulative proportion of adult overweight/obesity on the Y-axis. Earliest year of data collection and three-letter ISO country code call-outs.

Table S3: Eggers test on small-study effects for concentration indices

	Beta	SE of Beta	Z	P
Child Stunting	1.65	0.55	3.02	0.00
Child Wasting	-0.70	0.69	-1.02	0.31
Adult Overweight/Obesity	-1.50	0.61	-2.45	0.01

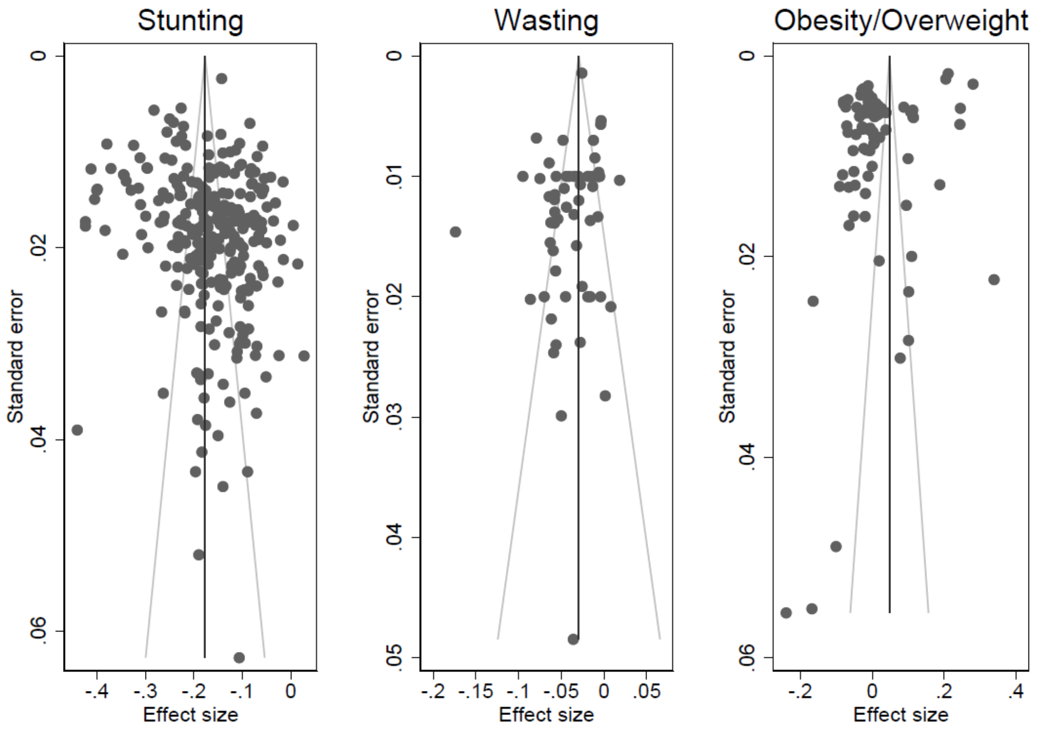

Figure S7: Funnel plots of concentration curves by outcome

Table S4A: Characteristics of multilevel studies measuring associations between economic inequality and nutrition prevalence

Author (publication year)	Survey years	Sample age (y)	Country	Sample size	Economic status measure	Outcome prevalence	Area level variable	Risk of Bias
**Child stunting**								
Reinbold (2011)*	2004	<5	Bangladesh	6908	DHS wealth Index	..	Region and district	Moderate
Reinbold (2011)*	2003	<5	Kenya	5767	DHS wealth Index	..	Region and district	Moderate
**Child Anaemia**								
van Deurzen et al (2014)*	2000-2011	<6	30 countries	152485	International Wealth Index Asset Score (DHS derived)	58.7%	Country	Moderate
**Adult underweight**								
Mandal (2007)	1992, 1997, 2002	<95	USA	448874	Not specified	..	State	Moderate
Subramanian et al (2007)	1998-1999, 1999-2000	15 - 49	India	77220	Expenditure	32.1%	State	Low
Hanandita and Tampubolon (2015)	2007	≥15	Indonesia	645032	Expenditure	14.4%	District	Moderate
**Adult overweight/obesity**								
Bjornstrom (2011)	2000-2001	..	USA	2875	Income	23%	Neighbourhood	Moderate
Zhang (2012)	2006	≥18	China	9586	Income	26%	Province	High

Author (publication year)	Survey years	Sample age (y)	Country	Sample size	Economic status measure	Outcome prevalence	Area level variable	Risk of Bias
Fan et al (2016)	2000, 2003-2008,	20 - 64	USA	10302	Income	34.4%	County and census tract	Low
Chang and Christakis (2005)	1990, 1996-1998,	≥15	USA	143931	Income	38.7%	Metropolitan Statistical Area	Moderate
Haithcoat et al (2019)	2014-2016, 2016	..	USA	954671	Income	31%	State	Moderate
Chen and Crawford (2012)	2000	≥18	USA	126298	Income	18.5%	State and county	Moderate
Kim et al (2006)	1974-1994, 1975-1998, 1990-1994, 1990, 1996, 1997, 1998, 2000	≥18	USA	181200	Income	19.5%	State	Moderate
Subramanian et al (2007)	1998-1999, 1999-2000	15 - 49	India	77220	Expenditure	6.2%	State	Low
Hanandita and Tampubolon (2015)	2007	≥15	Indonesia	645032	Expenditure	17.9%	District	Moderate
Mandal (2007)	1992, 1997, 2002	<95	USA	448874	Not specified	..	State	Moderate
**Adult (female) Anaemia**								
van Deurzen et al (2014)*	2000-2011	15 - 49	33 countries	373735	Wealth	40.2%	Country	Moderate

* These studies were not included in the meta-analysis

Table S4B: Variable adjustment for studies on association between economic inequality and nutrition prevalence

Author	Adjusted variables in main model
**Child stunting**	
Reinbold (2011)	Child age, sex, religion, mother's education, mother's employment status, mother's partner's education, mother's partner lives at home, mother's partner's age, mother's partner's age squared, household size, household size squared
**Child anaemia**	
van Deurzen et al (2014)	Household wealth index quintiles, survey year
**Adult underweight**	
Mandal (2007)	Restaurants, food stamp rates, unemployment rate, metro residency, age, education, children, race, marital status, work status, income, smoking, and health insurance.
Subramanian et al (2007)	Age, religion, caste, marital status, education, wealth, occupation, urban/rural, parity, smoking, drinking, tobacco chewing, tuberculosis, malaria, and state economic development.
Hanandita and Tampubolon (2015)	Age, gender, marital status, education, employment status, physical activity, household size, per capita expenditure, median per capita expenditure, and deprivation.
**Adult overweight/obesity**	
Bjornstrom (2011)	Age, gender, marital status, race, education, log family income, insurance, chronic conditions, smoking, and median household income.
Zhang (2012)	Gender, age, marital status, education, work status, occupation, wealth, income, province mean income, province mean education, urban and, province urban
Fan et al (2016)	Age, gender, marital status, family size, education, race/ethnicity, birthplace, income, survey year, census tract median income, and county median income.
Chang and Christakis (2005)	Age, household income, education, median income, population, and region.
Haithcoat et al (2019)	Inequality uniformity, median state household income, % insured, % on food assistance, age, gender, ethnicity/race, education, income, marital status, health insurance status, smoking, exercising, and alcohol.
Chen and Crawford (2012)	Marital status, age, income, education, employment status, race, poverty rate county, median income county, interaction of county gini: low income, interaction of state gini: low income, and state gini.
Kim et al (2006)	Age, gender, race, marital status, educational attainment, household income, state social capital, mean household income, and % black.
Subramanian et al (2007)	Age, religion, caste, marital status, education, wealth, occupation, urban/rural, parity, smoking, drinking, tobacco chewing, tuberculosis, malaria, and state economic development.
Hanandita and Tampubolon (2015)	Age, gender, marital status, education, employment status, physical activity, household size, per capita expenditure, median per capita expenditure, and deprivation.
Mandal (2007)	Restaurants, food stamp rates, unemployment rate, metro residency, age, education, children, race, marital status, work status, income, smoking, and health insurance.
**Adult (female) anaemia**	
van Deurzen et al (2014)	Household wealth index quintiles, and survey year.

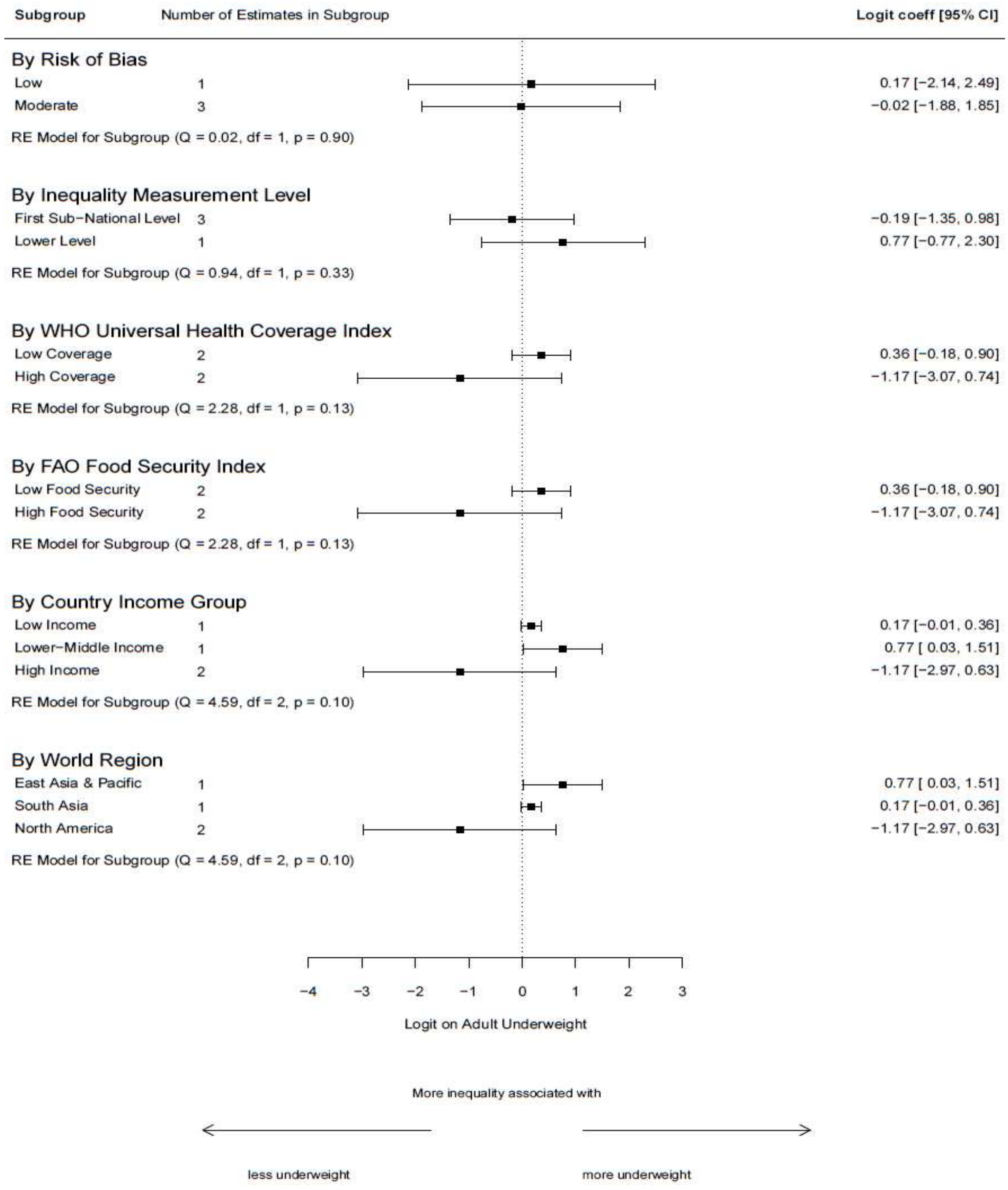

Figure S8: Subgroup analysis for associations between Gini coefficients and adult underweight

Logistic multilevel regression effects of Gini coefficient on a 0-1 scale on logged odds of malnutrition presented.

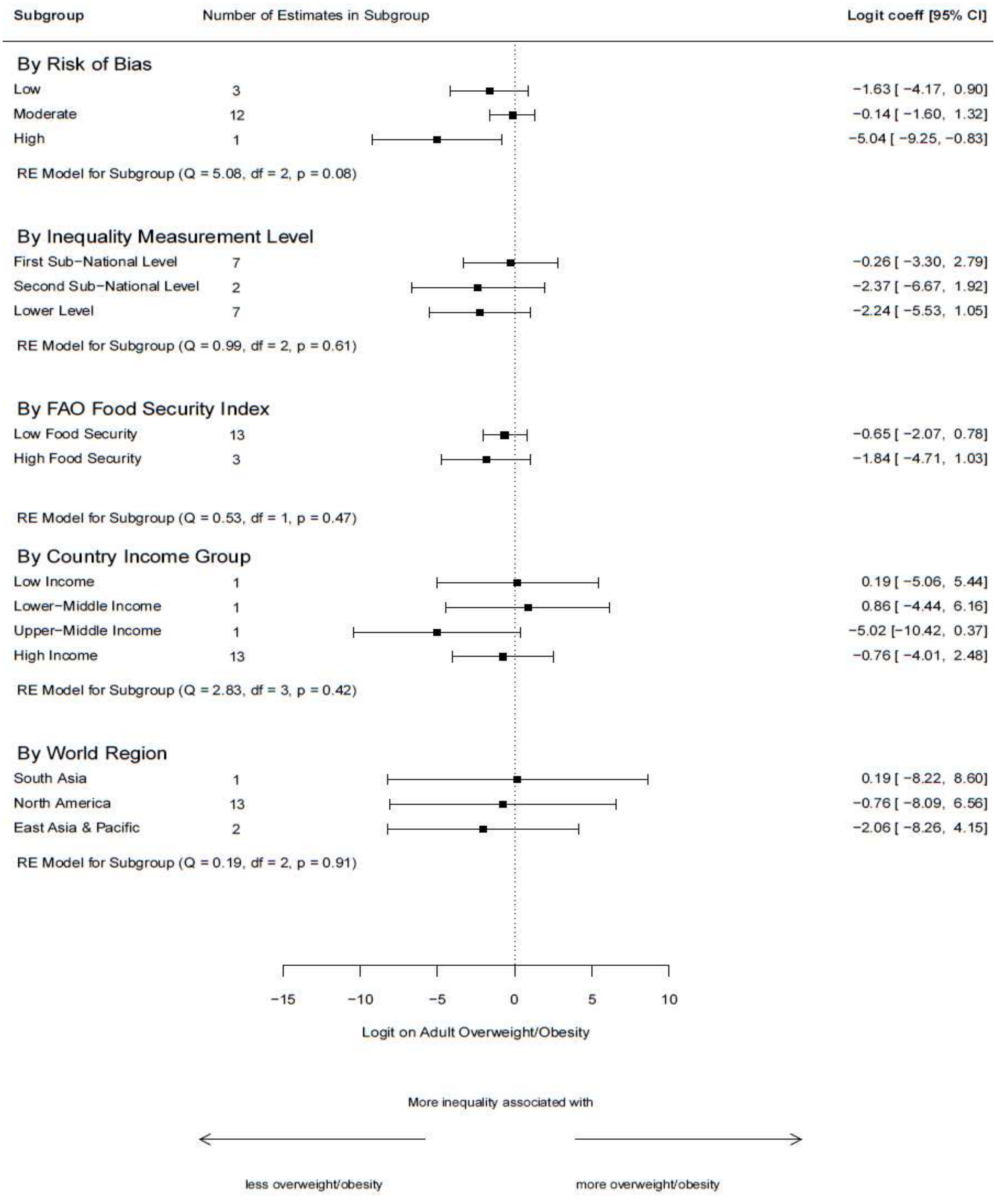

Figure S9: Subgroup analysis for associations between Gini coefficients and adult overweight/obesity

Logistic multilevel regression effects of Gini coefficient on a 0-1 scale on logged odds of malnutrition presented. Subgroup analysis by UHC Index omitted due to lack of variance.

Table S5: Eggers test on small-study effects for associations between Gini coefficients and nutrition outcomes

	Beta	SE of Beta	Z	P
Adult Underweight	-0.62	0.82	-0.75	0.45
Adult Overweight/Obesity	-1.23	0.55	-2.22	0.03

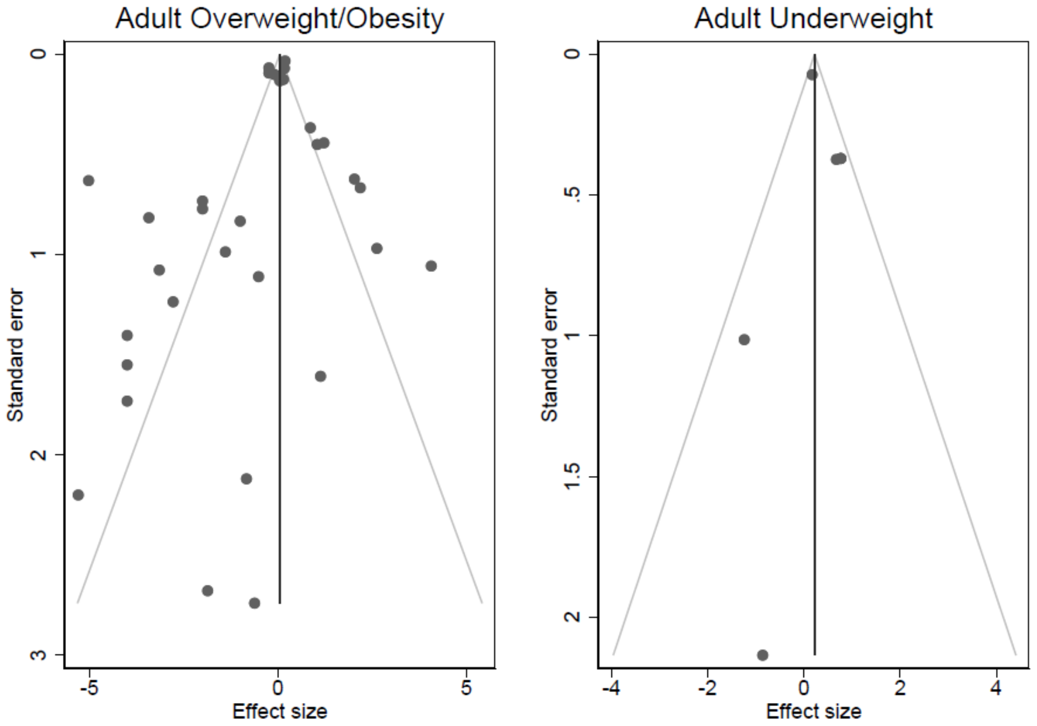

Figure S10: Funnel plots of studies on associations between Gini coefficients and nutrition outcomes
